# Genome Sequence of *Streptococcus phocae* subsp. *salmonis* Strain C-4^T^, Isolated from Atlantic Salmon (*Salmo salar*)

**DOI:** 10.1128/genomeA.01269-14

**Published:** 2014-12-11

**Authors:** Ruben Avendaño-Herrera, Rudy Suarez, Eduardo Lazo, Diego Bravo, Katerina O. Llegues, Jesús L. Romalde, Marcos G. Godoy

**Affiliations:** a Laboratorio de Patología de Organismos Acuáticos y Biotecnología Acuícola, Departamento de Ciencias Biológicas, Facultad de Ciencias Biológicas, Universidad Andrés Bello, Viña del Mar, Chile; b Interdisciplinary Center for Aquaculture Research (INCAR), Concepción, Chile; c Centro de Investigaciones Biológicas Aplicadas (CIBA), Diego de Almagro, Puerto Montt, Chile; d Facultad de Ciencias, Universidad San Sebastián, Puerto Montt, Chile; e Departamento de Microbiología y Parasitología, CIBUS-Facultad de Biología, Universidad de Santiago de Compostela, Santiago de Compostela, Spain

## Abstract

*Streptococcus phocae* subsp. *salmonis* is a fish pathogen that has an important impact on the Chilean salmon industry. Here, we report the genome sequence of the type strain C-4^T^ isolated from Atlantic salmon (*Salmo salar*), showing a number of interesting features and genes related to its possible virulence factors.

## GENOME ANNOUNCEMENT

*Streptococcus phocae*, a beta-hemolytic, Gram-positive bacterium and member of the pyogenic streptococcal group (1), was first isolated from clinical specimens of harbor seal (*Phoca vitulina*) (2), and then recognized as an important pathogen for different seal species in several countries, causing pneumonia or respiratory infection (3–6). From 2005, the bacterium has been identified from disease outbreaks in Atlantic salmon (*Salmo salar*) cage farmed in Chile (7). Fish infected showed exophthalmia with the accumulation of purulent and hemorrhagic fluid around the eyes, ventral petechial hemorrhages, and skin abscesses (8). Although initially *S. phocae* was included as a member of the warm water streptococcosis, Yáñez et al. (9) demonstrated that *S. phocae* caused mortality of Atlantic salmon farmed below 10° C, allowing us to extended the known geographic range of this fish pathogen.

Until now, *S. phocae* isolates from Atlantic salmon have demonstrated a biochemically, antigenically, and genetically homogeneous taxon (8, 10), a very distinct group from the seal isolates, including the type strain ATCC 51973^T^ (2, 5), but so far scarce information has been available about the pathogen and virulence mechanisms. González-Contreras et al. (11) suggest that the surface hydrophobicity and binding capacity to fish cells, as well as the effect of the live cells of *S. phocae*, play important roles in the pathogenicity of streptococcosis at an early stage in the disease process, in which the pathogen needs to possess the ability to overcome the antibacterial activity of the fish serum. Moreover, the survival of *S. phocae* in mucus could be relevant *in vivo* to facilitate the colonization and subsequent invasion of the host (12). No commercial vaccine is available, and attempts to control of streptococcal infection by oral treatments using florfenicol, erythromycin, and oxytetracycline have met with various degrees of success (13).

Recently, a polyphasic study was undertaken to clarify the taxonomic position of *Streptococcus phocae* strains isolated from Atlantic salmon in Chile (14). Microbiological, genetic, and molecular results demonstrated that these isolates represent a novel subspecies of *S. phocae*, for which the name *Streptococcus phocae* subsp. *salmonis* was proposed, and the type strain C-4^T^ (14).

The genome of *S. phocae salmonis* C-4^T^ was sequenced from a DNA library using a 454 platform (GS-FLX, Roche) with 21-fold-coverage. The 454 reads were *de novo* assembled in 105 contigs using an assembly pipeline with Newbler 2.7 (Roche Diagnostics Corporation) and CLC Genomics Workbench 2.0.7 and then reassembled with CAP3 software (15). The estimated length of the chromosome is 1,659,206 bp, with a CG content of 36.74%, containing a total of 1,685 predicted open reading frame (ORF) regions. Analysis of the whole genome of C-4^T^ revealed sets of genes related to colonization, biofilm formation, invasion, and destruction of the host tissues, such as hemolysins, collagen adhesion protein, and capsule biosynthesis proteins (11), as well as particular metabolic properties related to the stress response and long-term survival outside the host. This genome sequence can facilitate comprehensive bioinformatic and phylogenetic analyses aimed toward adoption of preventive approaches to combat this pathogen.

### Nucleotide sequence accession numbers.

This whole-genome shotgun project has been deposited at DDBJ/EMBL/GenBank under the accession number JSAP00000000. The version described in this paper is version JSAP01000000.
